# Synovial Osteochondromatosis at the Carpometacarpal Joint of the Thumb

**DOI:** 10.1155/2017/3974342

**Published:** 2017-01-11

**Authors:** Satoru Yonekura, Hiroyoshi Hagiwara, Takahiro Nishimura, Hiroshi Amagai, Mayu Yamamura, Osamu Miyamoto, Sueo Nakama

**Affiliations:** ^1^Department of Orthopedics, Tochigi Medical Center Shimotsuga, Ohira-machi Kawatsure 420-1, Tochigi City, Tochigi 329-4489, Japan; ^2^Department of Orthopedics, JCHO Utsunomiya Hospital, Minamitakasago-cho 11-17, Utsunomiya, Tochigi 321-0143, Japan

## Abstract

Synovial osteochondromatosis (SOC) is a benign tumor characterized by synovial connective tissue metaplasia. SOC commonly affects major joints including the knee followed by the hip, elbow, and wrist. SOC cases in the hand are not reported as often as SOC of major joints. Particularly SOC of the carpometacarpal joint of the thumb is rare. We report on a 57-year-old female with primary SOC of the carpometacarpal joint of her left thumb. Surgical excision was performed and the patient had no symptoms with full range of motion of her left thumb. At 3 years of follow-up, there was no recurrence.

## 1. Introduction

Synovial osteochondromatosis (SOC) is a benign neoplasm characteristic of synovial connective tissue metaplasia within the synovial membranes of joints, tendon sheaths, or bursae [1–3]. SOC commonly affects all major joints including the knee, followed by the hip, elbow, wrist, ankle, and shoulder joints [1, 4, 5]. SOC in the hand is rare. The most cases of reported SOC in the hand were SOC of the wrist and flexor tendon of the finger [3]. Herein, we report on a 57-year-old female with primary SOC of the carpometacarpal (CM) joint of her left thumb.

## 2. Case Presentation

**Figure 1:**
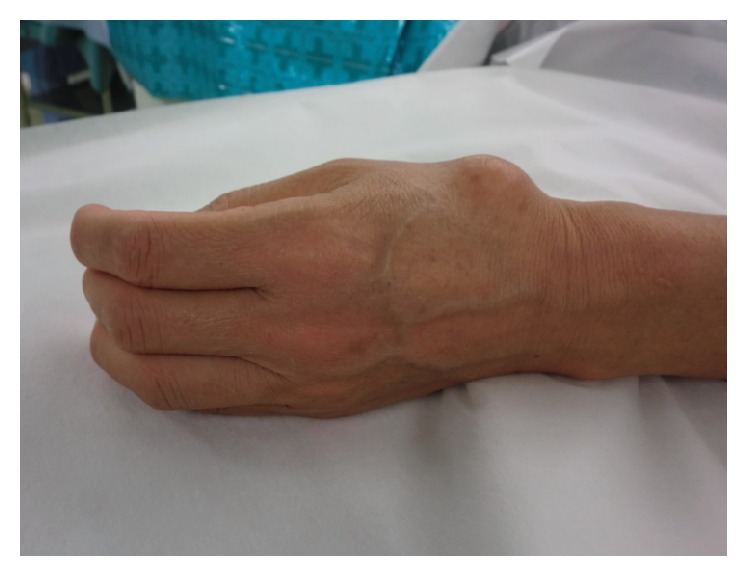
A palpable and tender mass, approximately 2.5 cm × 2.5 cm × 1.5 cm in diameter on the lateral aspect of the left thumb.

A 57-year-old woman was present at our institution with a chief complaint of the pain and swelling at the left thumb. She had the pain for 5 years and noticed the swelling 3 years ago. She had no history of trauma and no other relevant medical history. On physical examination, the patient had a palpable and tender mass, approximately 2.5 cm × 2.5 cm × 1.5 cm in diameter, located over the dorsal aspect of the CM joint of the left thumb without involvement of the skin (Figure 1). Results of laboratory analyses were normal.

**Figure 2:**
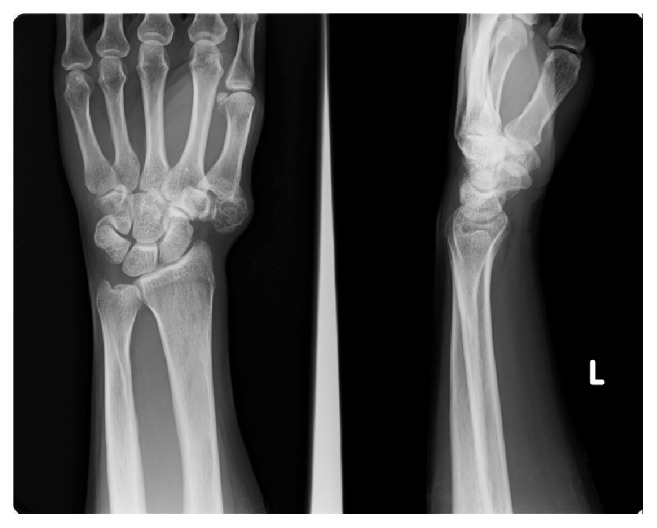
PA and lateral plain X-ray of the left hand showing a soft tissue lesion with calcifications adjacent to trapezium and the base of the first metacarpal bone.

**Figure 3:**
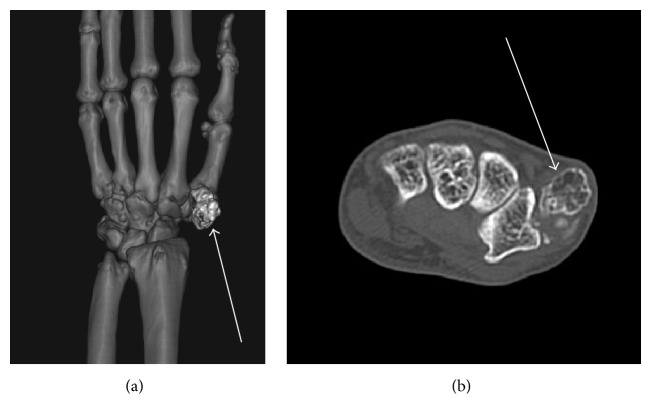
(a) Preoperative CT imaging of the left hand showing a nodule (white arrow) adjacent to trapezium and the base of the first metacarpal bone. (b) Axial section of CT imaging showing the tumor (white arrow).

A plain X-ray of the hand revealed mild soft tissue swelling and radiopaque bodies around the CM joint of the left thumb without erosion of the base of the first metacarpal bone and trapezium (Figure 2). Computed tomography (CT) imaging showed a 2.1 cm × 1.9 cm × 1.4 cm nodule (Figure 3). Due to hospital limitations, magnetic resonance imaging (MRI) was not obtained.

**Figure 4:**
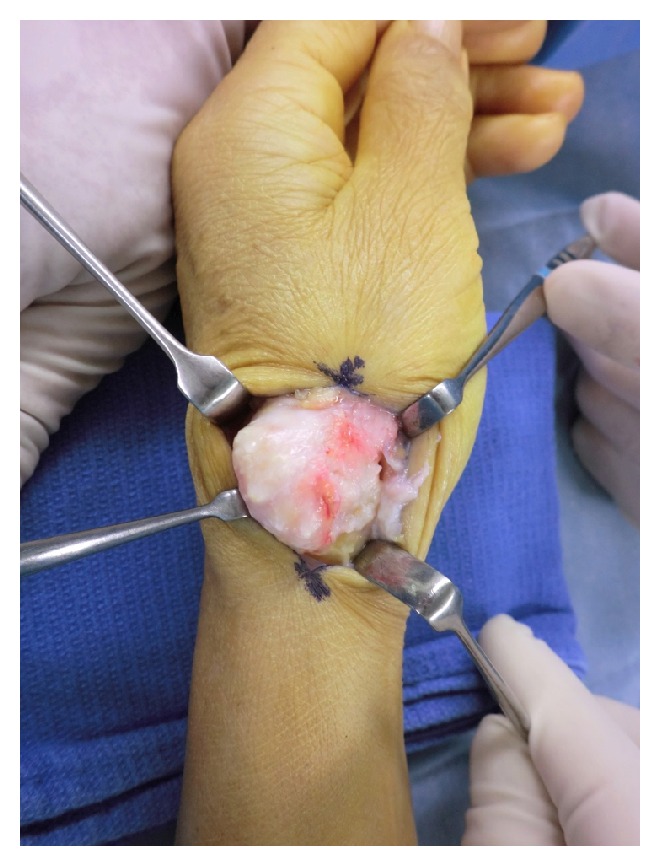
An intraoperative photograph showing marginal incision of the tumor.

**Figure 5:**
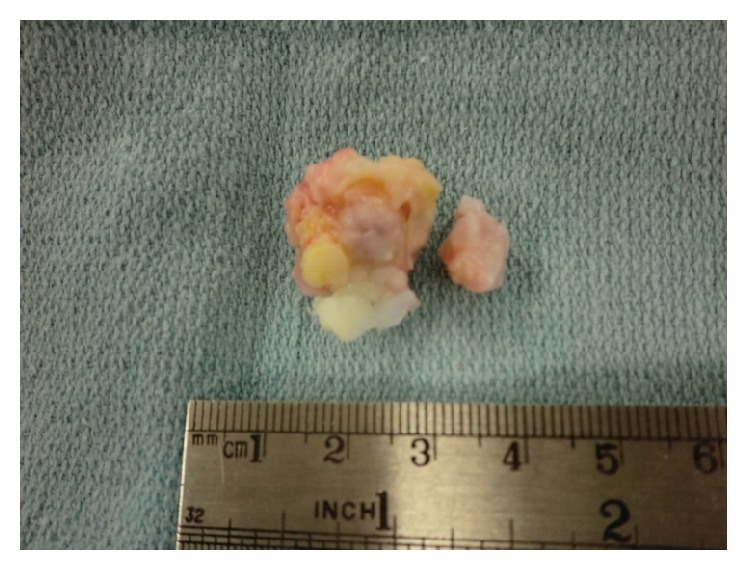
Gross pathological specimen of excised tumor.

**Figure 6:**
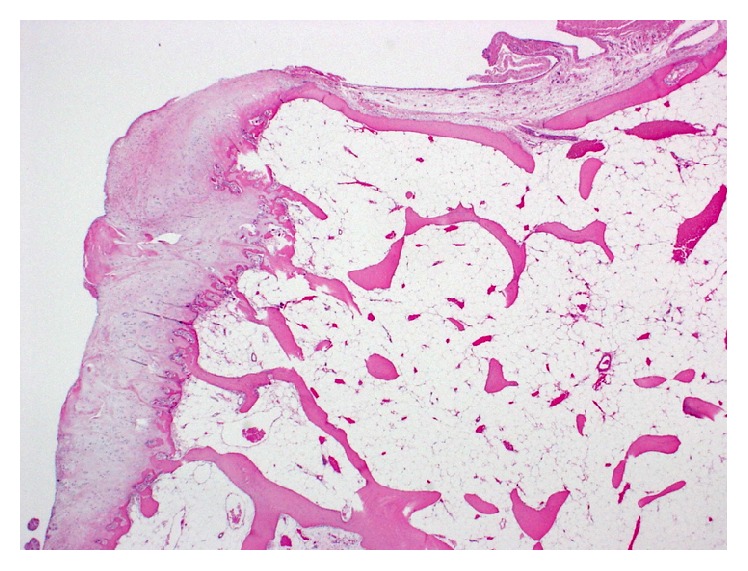
Histopathology of resected tumor showing cartilaginous tissue with calcification (hematoxylin and eosin stain ×4).

Marginal resection of the tumor with synovectomy was performed after the CM joint capsule was incised (Figures 4 and 5). Pathology examination revealed fibrous tissue containing cartilaginous areas with calcification, which was consistent with SOC (Figure 6). There was no evidence indicating malignancy.

There is no evidence of recurrence and the patient had a return of full range of motion of her left thumb 3 years postoperatively.

## 3. Discussion

This was a rare case of SOC in the CM joint of the thumb. SOC is a benign tumor characterized by a metaplasia of the synovial membrane resulting in an accumulation of intra-articular cartilaginous nodules [1–3]. The etiology is still unclear. Milgram proposed 3 histological phases based on the maturation of the lesion: (i) active intrasynovial disease without loose bodies; (ii) transitional lesions with both active intrasynovial proliferation and free loose bodies; and (iii) multiple osteochondral bodies with no intrasynovial disease [6].

The most common articular lesion of SOC is the knee followed by the hip and elbow [1, 5]. SOC in the hand is rare compared to SOC of large joints. Published cases of SOC in the hand were mostly SOC of the wrist and tendons or joints of the finger [3]. Among joints in the hand, interphalangeal and metacarpophalangeal joints are the most common affected sites [3]. To the best of our knowledge, there is only one English literature of SOC in the CM joint [7].

Symptoms of SOC are nonspecific, including a palpable mass, swelling, pain, tenderness, and restricted movement of the joint [1, 4]. Complications of SOC include secondary degenerative osteoarthritis [3]. Peripheral nerve compression syndrome caused by SOC was reported [8]. Radiographic features of SOC depend on the maturity of the tumor [1]. CT imaging or MRI can detect SOC at the initial stage because radiographs may lack the ability to visualize features of immature SOC such as an effusion and noncalcified loose bodies [1, 9]. Matured SOC has superficial bone erosions and calcified loose bodies [1, 10].

For making a diagnosis histological examination is necessary. The common characteristics of SOC on gross pathology are synovium with multiple nodules. SOC on microscopic examination is known to have focal and circumscribed areas of hyaline cartilage embedded within synovial connective tissue [3]. The differential diagnosis of SOC in the hand includes chondrosarcoma, rheumatoid arthritis, chronic infection, trauma (osteochondral fracture), osteochondritis dissecans, osteoarthritis, neuropathic arthritis, and gout [3]. SOC is unlikely to transform into malignant synovial chondrosarcoma [2, 11] and all cases of malignant transformation were in large joints [2, 12]. The risk of malignant change is reported to be 5% [12].

The most effective treatment is synovectomy with removal of loose cartilaginous nodules [13, 14]. Recurrence after resection is most likely due to inadequate excision [15]. The recurrent SOC indicates additional surgery [1].

## 4. Conclusion

Synovial osteochondromatosis in the carpometacarpal joint of the thumb is rare. Surgical excision and histopathological examination are necessary for diagnosis.
